# A New Input Device for Spastics Based on Strain Gauge

**DOI:** 10.3390/s17040880

**Published:** 2017-04-17

**Authors:** Niels Buchhold, Christian Baumgartner

**Affiliations:** Institute of Health Care Engineering with European Testing and Certification Body of Medical Devices, Graz University of Technology, Stremayrgasse 16/II, 8010 Graz, Austria

**Keywords:** tactile sensors, assistive technologies, power wheelchair, medical systems, robotic, joystick, strain-gauge, spastic, spasticity

## Abstract

This article presents a new sensor for use by people with spastic disorders and similar conditions and enables them to steer and control medical devices such as electric powered wheelchairs. As spastic patients often suffer from cramping of their extremities, which can then no longer be controlled, using a standard joystick while operating a powered wheelchair can lead to dangerous situations. To prevent this, we designed a sensor based on strain gauges, which is shaped like a flat disc that can be operated using any body part. By shifting weight along the *x*- and *y*-axis, the disc tilts in all directions thereby generating proportionate output signals. The disc can also be pressed downward (*z*-axis), for example, to open a wheelchair’s menu. Thanks to the sensor’s flat disc-like construction and the option of mounting it into a control panel, users are not in danger of becoming stuck on the disc during spastic episodes. In the event of a spasm, body parts simply slide over the disc reducing risk of unintended actions. The sensor is adaptive and adjustable enabling it to fit a user’s range of strength and motion at any time. It was developed to ensure users can operate sensitive systems safely.

## 1. Introduction

The role of sensors as interfaces between man and machine in today’s world is gaining in importance. Joysticks and joystick-like sensors can be operated almost intuitively and are commonly used in a variety of control systems as input devices. Fields of application such as medical technology require the highest level of safety for operation. Using a sensor [1] (joystick) is problematic especially for physically disabled people suffering from spasms, as the range of motion (strength and hub) typically undergoes constant spasms. Cramp-like movements while using sensitive systems such as powered wheelchairs can result in uncontrollable and dangerous situations. Moreover, users are at risk of injury if their hand cramps around a joystick causing excessive strain on bones and tendons [2]. 

Therefore, the first task during development was to ensure the sensor had the proper ergonomics to prevent injury. To accommodate various medical conditions and the very different ranges of motion associated with them, a sensor should be adapted to each user. Making adjustments to a conventional sensor would be very costly and necessary for each progression of a medical condition. Moreover, for some medical conditions the success of such modifications would be only temporary because users’ range of motion undergoes constant change. For that reason, the second task during development was to design the sensor in such a way that inhomogeneous forces (i.e., the maximum force a user can apply depending on the direction) can be converted into homogenous output signals. The goal in doing so was to generate consistent output signals for each direction despite varying degrees of force to drive an electric powered wheelchair, for instance, in each direction at the same speed. A computer mouse could also move a pointer at the same speed on a screen in each direction. This development task, however, requires that the sensor be capable of processing a range of force between 0.05 N and 25 N. 

When operating an electric powered wheelchair, new control systems such as eye tracking, voice control and brain-computer interfaces [3,4,5,6] are not usable for people with spastic conditions due to safety reasons. The reason is that the uncontrolled contraction of various muscle groups would cause the triggering of unintended actions. Tongue controls [7,8] are suitable for spastics to a limited degree since the tongue is affected only in rare cases. The aforementioned input methods make precise and complex control actions difficult as each change of direction or speed requires a new command. Moreover, eye tracking is affected by natural eye reflexes and completely unsuitable for safety critical control tasks. Voice control can usually only process one command at a time and is unreliable in loud environments [9]. 

**Figure 1:**
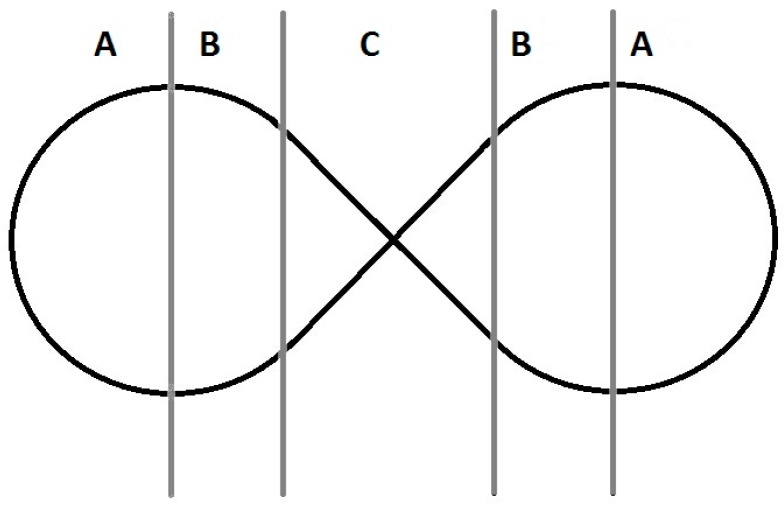
“Form 8” obstacle course.

In order to measure the quality and opportunities of sensor-based control, we developed a simple yet effective test during our research. The user is asked to navigate through an obstacle course with the electronic power wheelchair (EPW) (see Figure 1) or is tasked with moving a mouse pointer around a screen and tracing a shape.

The obstacle course consists of three areas with different requirements for specific input movements. While in area A the user is asked to drive in a consistent radius, in area B the radius of the curve changes and gradually transitions into a straight section in area C. This test includes all variations of possible changes in direction. For an increased level of difficulty, the obstacle course can be performed in reverse as well. During the tests, an acceleration sensor and a video camera are attached to the EPW. A sensor will have optimal effectiveness if it records only small jolts and spikes in direction and speed. A review of the pictures taken by the camera shows the degree of precision to which the line was traced. With digital input devices or voice control, it is not possible to follow the line through the obstacle course at a consistent speed. In area A and B the route driven would vary because the radius would be constantly adjusted. A consistent drive is only possible with a proportional sensor. 

Until now, proportional sensors, such as joysticks, could only be used to a limited degree by people with spastic conditions due to the safety risk in case of cramping. Obstacle detection systems that helps users navigate [10,11] make sense but they are expensive. With these assistive systems, a user cannot complete an entire trip independently because the navigation aid takes over automatically when obstacles are encountered. After surveying users, we found that assistive systems often intervene in that desired route, making them more or less undesirable as aids. Control systems that record spastic movements over certain period [12] and use an algorithm with an averaging function to generate the next probable direction a user wants to move toward, could be combined with the strain-gauge disc presented here. But even this would hinder direct and independent control by users. Users only accept direct controls for wheelchair rides that take place in very narrow areas or for sporting activities such as EPW hockey. 

US patents [13,14,15] describes input devices with strain-gauges (SG) that are designed like conventional joysticks. The method of determining positions with the help of strain gauges is therefore commonly known [16]. The innovative aspect of this strain-gauge disc as compared to other sensors, their design and how they determine positions is listed below:

1. The sensor’s flat disc-like design.
2. The construction and shape of the movement carrier constructed using carbon fiber reinforced composite (CFRP).
3. The movement carrier’s highly precise ability to return to its original position after being deflected.
4. The software algorithm designed to adapt the sensor to a user’s own range of strength and motion.
5. The differential processing of measurements for plausibility checks to increase user safety.
6. The sensor’s range of sensitivity between 0.05 N and 25 N.
7. The sensor’s unsusceptibility to excess strain (e.g., in case of spasms, it withstands loads up to 1400 N).

**Figure 2:**
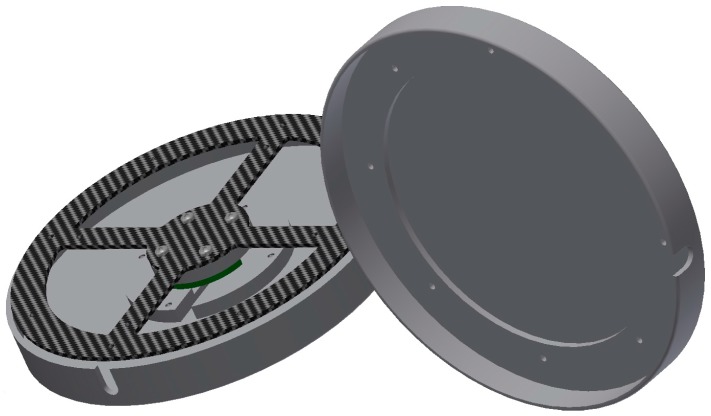
Strain-gauges device (SGD) drawing.

**Figure 3:**
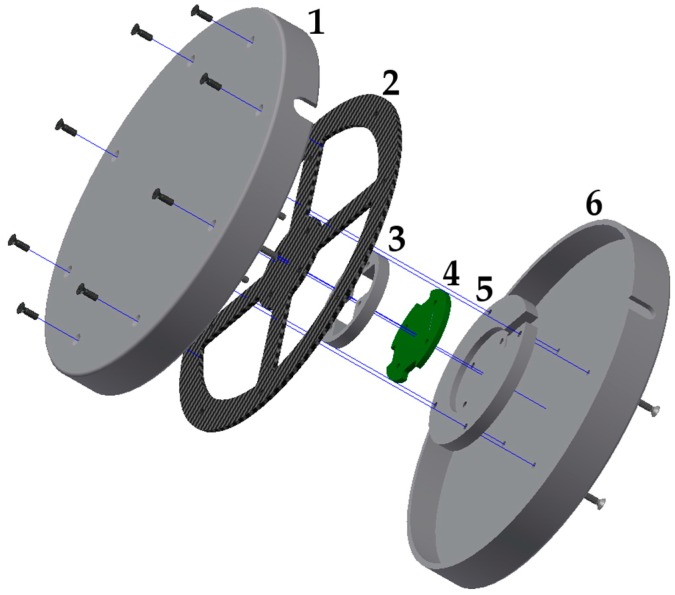
Exploded View of SGD.

All of the features mentioned here were implemented in this development and are described in this article. This includes a description of the prototype’s hardware (Figure 2 and Figure 3) as well as a schematic look at the software and its algorithms.

## 2. Hardware of the SGD

### 2.1. Basic Construction

Because the sensor must sometimes undergo very high strain as in the case of spastic episodes, its construction needs to meet stability criteria. The strain gauge disc—SGD’s maximum load capacity is 1400 N. This load limit can be increased by using a more sturdy housing (see Figure 3, Part 1 and Part 6), if needed. Despite the high load-bearing capacity, the sensor must be capable of processing a user’s fine-motor movements since users with spastic disorders have a similar range of strength and motion as healthy people. It is very important that users do not trigger unintended actions during spastic fits. A flat disc (120 mm in diameter) seemed to make the most sense due to the aforementioned reasons. The actual sensor (see Figure 3) is made of a carbon fiber reinforced composite (CFRP) carrier (Figure 3, Part 2) that is attached to four strain gauges. A circuit board is positioned underneath the carrier (Figure 3, Part 4) with four differential amplifiers AD 623 [17] and an analog-to-digital converter (ADC) AD 7811 [18] with an integrated SPI (Serial Peripheral Interface) data output. The user can incline the disc in any direction by shifting its weight (*x*-, *y*-axis) on the upper cover (Figure 3, Part 1). As a result of the design, the edge of the CFRP carrier is pressed downwards during *x*- and y-axis movements, while the opposite side of the CFRP carrier lifts up. Due to this fact, a plausibility test can be carried out since a defined measured value of the opposite strain gauge must be present for each measured value. This software process is described in detail in Section 3.2. The upper housing (Figure 3, Part 1) can also be pressed down. In doing so, all four strain gauges are deflected in one direction (*z*-axis), which makes other control options possible, such as operating a computer mouse (mouse click). The sensor’s parts labeled Part 3 and Part 5 (Figure 3) only serve as spacers. The entire sensor can also be integrated into a control panel in front of the user, such as in an electric powered wheelchair. In this case, for example, the entire control panel could be made of CFRP. The interlocking parts of the housing (see Figure 3, Part 1 and Part 6) protect the CFRP carrier against damage.

The SGD (see Figure 3) consists of the following components. Part 1 and Part 6 make up the upper part of the bottom casing. The CFRP movement carrier (Part 2) is screwed to the upper casing (Part 1). The circuit board (Part 4) is positioned between the spacers (Part 3 and Part 5) and soldered to the strain gauge. 

### 2.2. CFRP Carrier

**Figure 4:**
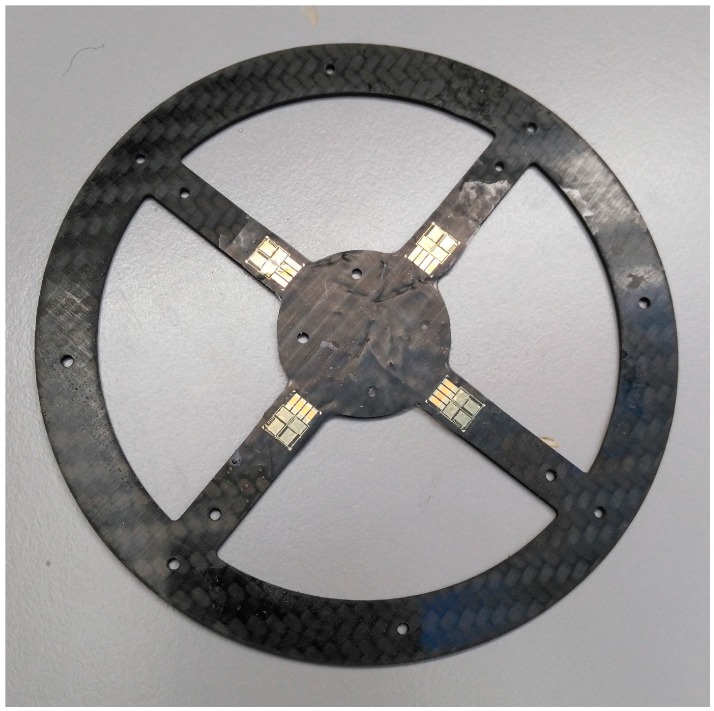
CFRP carrier with affixed strain-gauges.

**Figure 5:**
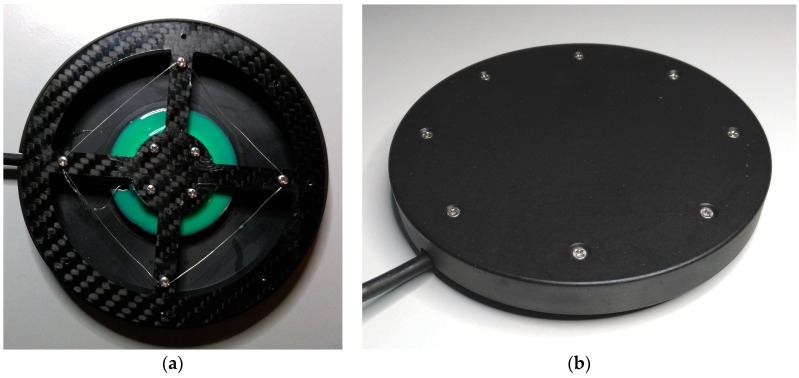
SGD without upper cover (**a**) and with cover (**b**).

The prototype’s CFRP carrier (Figure 4) is made of a CFRP plate measuring 1.3 mm in diameter. The material thickness can be adjusted depending on the application as CFRP has excellent durability compared to other materials. Compared to aramid fiber reinforced composite (AFRP) and glass fiber reinforced composite (GFRP), CFRP has superb dynamic properties [19]. The CFRP’s reset behavior is of particular importance. When the sensor is overstrained, the interlocking parts of the casing (see Figure 3, Part 1 and Part 6) protect the CFRP carrier against damage. Thanks to the housing’s design, the maximum stroke of the sensor can be mechanically limited. If the load is too high (max. 1400 N) or the stroke is too forceful, the incoming forces are distributed past the CFRP carrier via the housing. The CFRP compensated strain gauges are glued and soldered to the circuit board. Additional cross linking silicon can then be poured over the circuit board and the strain gauges. This makes the sensor usable in humid environments. For testing purposes, the CFRP carrier was also subjected to stretching. The design shown in Figure 5, which has a CFRP material thickness of 1.3 mm, broke under a tensile load of 102 N. It is not possible, however, to pull the sensor’s housing without any aids. The 1.3 mm material thickness is a very good compromise between responsiveness and durability.

### 2.3. Circuit Board and Microcontroller

**Figure 6:**
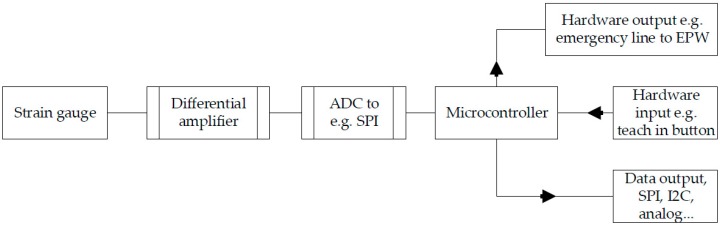
Schematic sensor hardware.

On the circuit board (Figure 3, Part 4) there are four differential amplifiers AD 623 [17], a 10-bit ADC AD 7811 [18] and a voltage stabilizer. The differential amplifiers’ gain is set by an external resistor. All four values (see Figure 6) are constantly transmitted by the ADC via an SPI interface (slave) to a downstream microcontroller SPI (master). The microcontroller then generates the desired output signals for the hardware. The microcontroller’s I/O pins, which are also connected to the downstream microcontroller, would activate in case of a malfunction. This makes it possible to initiate emergency measures.

### 2.4. Different Versions

During development three different versions of the sensor were constructed for people with spastic conditions. Users can decide which version is most suitable for them. 

Version 1: The CFRP carrier and its shaping would be adjusted to meet the requirements. The material thickness and the arrangement of the CFRP layers would be taken into account. The shaping reinforces stability and lends flexibility to the movement carrier. 

Version 2: Setup is the same as version 1. In addition, the CFRP carrier is cast in an addition-crosslinked, thermally vulcanizing silicone rubber. The damping properties will vary depending on the Shore-hardness. Other damping materials, such as compression springs or other polymers, did not bring about the desired effect because the thermal expansion coefficients influenced the CFRP carrier in a counterproductive manner.

Version 3: The entire control panel of an EPW is made of CFRP. The strain gauges are attached to the panel using adhesive. Corresponding CNC milling patterns around the strain gauges influence the sensor’s sensitivity.

During the series of tests, CFRP carriers with material thicknesses of 0.45 mm to 2 mm were tested. Thanks to the shaping, the sensitivity and the maximum load in regard to pulling in particular can be adapted. 

## 3. Sensor Operations

### 3.1. Basics

If external forces act on the CFRP carrier (see Figure 3, Part 2) from outside the housing top (see Figure 3, Part 1), its tilt changes slightly. As a result, the opposite strain gauge is deflected differentially. The differential values that result are added together in the downstream controller, which then increases the resolution. Using a more powerful ADC would increase the resolution accordingly.

**Figure 7:**
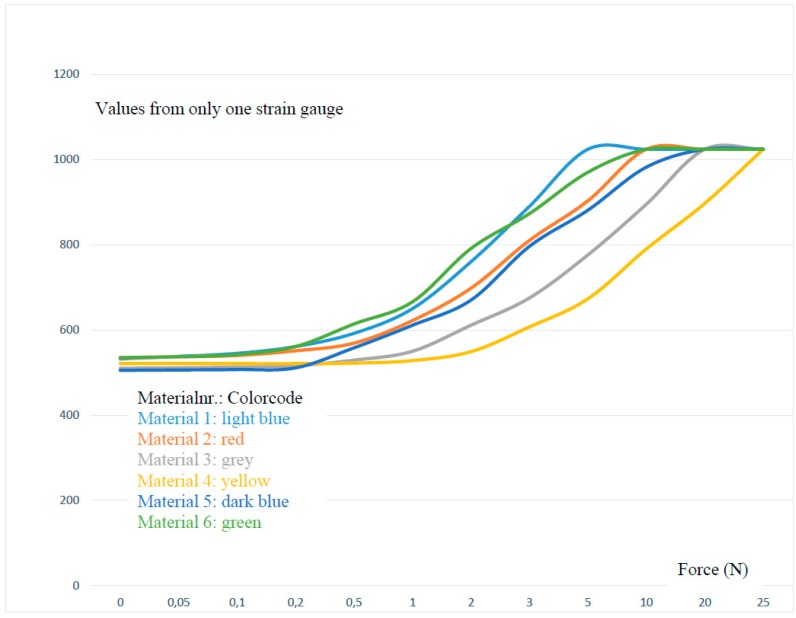
Visualized, measured values from Table 1.

Example Calculation at the time of t = 1 (values dimensionless): 

$X_{Neutral}=512$Measured neutral value;$X_{Right\;t=1}=640$Measured value right strain gauges at t = 1;$X_{Left\;t=1}=430$Measured value left strain gauges at t = 1;$X_{t=1}=|X_{Neutral}-X_{Right\;t=1}\left|+|X_{Neutral}-X_{Left\;t=1}\right|$$X_{t=1}=210$Output x-value to the microcontroller;

Depending on the version, the restoring force is caused by the CFRP on its own or additionally via silicone rubber. Table 1 and Figure 7 show the measured values of one SG in relation to the applied force. Various silicone rubber mixtures and different material thicknesses (see Table 2) are shown by way of example. Due to the fact that the measurement curves are identical in each direction, the measurement was carried out only for one SG. Depending on the CFRP motion carrier and the polymers shore hardness, the maximum value of the 10-bit ADC (1024) is achieved at different degrees of force. Additional forces impact the housing beyond the maximum value of 1024 (see Figure 3, Part 1 and Part 6).

### 3.2. Plausibility Check

The plausibility check constantly examines the values recorded by the sensor during operation. Directly after production, an initialization process is carried out. Under various loads, all of the strain gauges’ possible measured value combinations are stored during this phase. Under normal operating conditions, the measured values of the opposite strain gauge are compared to the previously registered value combinations. If the values deviate from the previous values, then there is an error. 

This makes it possible to detect many errors or damage to the hardware immediately. A specific value is documented if a strain gauge loses contact with the CFRP carrier. The specific value of the strain gauge opposite that one is searched for. If the pair of values do not match, then there must be an error. Measures are then taken to protect the downstream system from malfunctions (see also Figure 6). Breakage also causes the currently measured value pair to not match a stored pair of values. All of the errors that occurred during the test phase were immediately detected by the plausibility check. In addition to breakage tests, the sensor was also immersed in water. 

### 3.3. Individual Adjustment of the SGD

**Figure 8:**
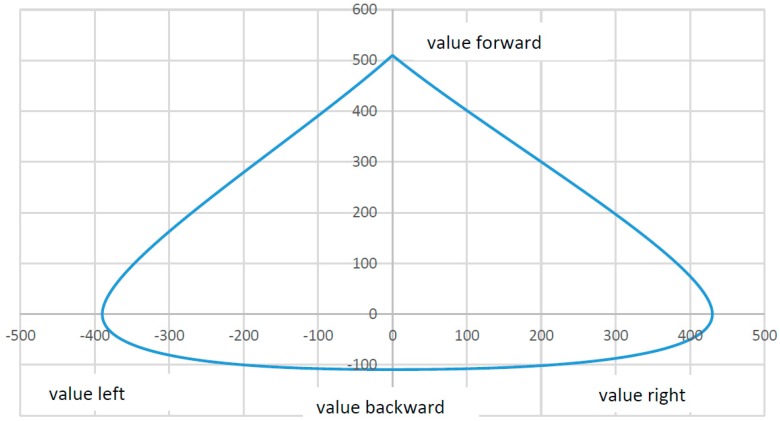
Force Curve (force to value), max. value from neutral position for Material 3 (see Table 3).

Due to the fact that symptoms vary from patient to patient, the sensor needs to be adapted to a user’s own range of strength and motion. Conventional sensors, such as joysticks, have a certain accuracy (resolution), a certain amount of force required for deflection, and a certain stroke to overcome the necessary paths by a fixed amount. As described in the introduction, certain groups of physically disabled people are unable to use standardized joysticks for a variety of reasons. This issue has to do with this group of users’ range of motion (force and stroke). Moreover, the existing range of motion is affected by outside influences such as ambient temperature [20]. A conventional sensor would have to undergo mechanical adjustments on a constant basis, which would be quite inconvenient. To adapt the sensor described here to the user’s range of strength and motion, the SGD is moved once in each direction in a circular motion. During this 10-second learning process, the maximum x and y coordinates are stored. When vertical pressure is applied to the top of the housing (Figure 3, Part 1), the maximum values for z-axis can be stored as well. The absolute zero position or resting position, is set during a calibration process for each SGD after assembly, in conjunction with the initialization process (see Section 3.2 plausibility check). This eliminates excess production costs since production tolerances are relatively insignificant. To serve as a reference, a patient’s strength and stroke were documented. The result in Figure 8 shows an inhomogeneous progression of force applied in different directions.

When a reference was established previously (deflection and force in relationship to the measured value), Figure 8 can be used to determine the force applied by the patient. In this test, the patient was able to apply the following maximum deflections, (force) without taking the z-axis into account:

If the values in Figure 8 were sent directly to an EPW, the user would only be able to drive forward, right, and to the left at an acceptable speed. The maximum values for backwards are insufficient to move the wheelchair. The correct multiplication factors can help attain a consistent output signal.

Example: 

The factor for a 10-bit ADC is calculated with this formula:

$$\frac{MaxValueADC}{MaxPatientMeasuredValue}=factor$$

$$\left(Sample\;calculation\;forward\right)\;\frac{1024}{510}=2,007$$

All of the values documented during operation have to be converted by corresponding factor in Table 4 to attain a consistent output signal. 

Example Calculation: 

(Right value from Table 3; 10-bit ADC)

**Figure 9:**
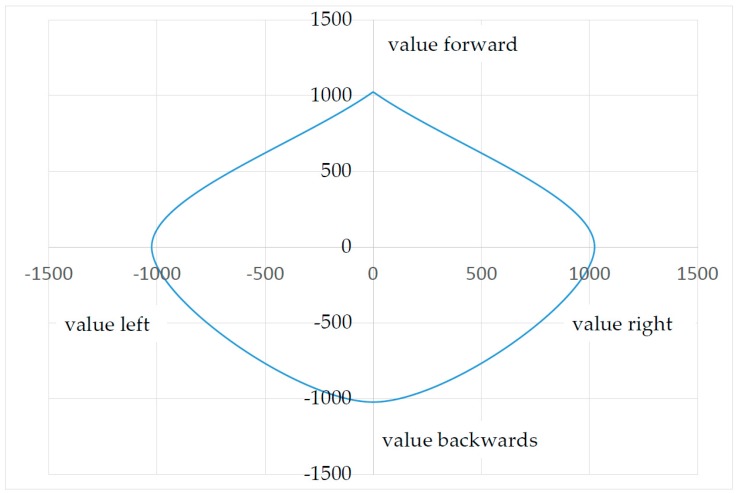
Maximum output data curve.

$X_{MaxRight}=430$Maximum right direction;$X_{FactorRight}=2,381$Calculated factor see Table 3;$X_{RightExample}=200$Example value to the right;$X_{OutRight}=X_{RightExample}*X_{FactorRight}$Digital output value;$X_{OutRight}=476$Digital output;

These output values are then offset by the differential output value of the opposite strain gauge, as seen in the example calculation (see Section 3.1 Basics). At this time the plausibility check (see Section 3.2 Plausibility check) is also carried out. Using the algorithm shown above and its multiplication factors, the measured values of the input forces (see Figure 8) are converted to generate corrected values for the downstream systems. If the user applies his or her previously set maximum force, the output values reach the maximum value in each direction (see Figure 9). The user is thus able to move a computer mouse or an electric powered wheelchair in every direction at the same speed, despite different input forces.

### 3.4. Software

**Figure 10:**
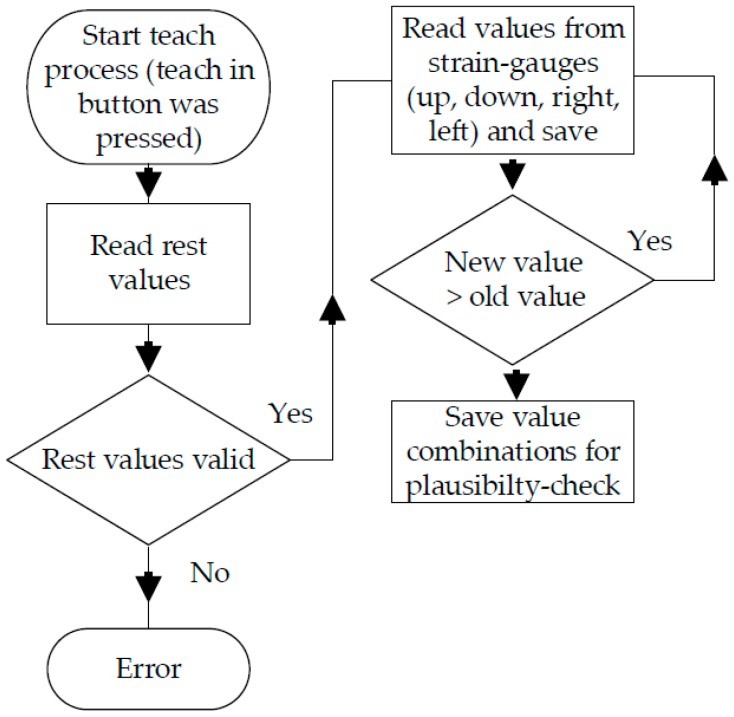
Schematic sensor software teach-in process.

The software procedures are divided into two separate processes. In order to adapt the strain gauge disc to a user (see Figure 10), the teach-in button (see Figure 6) must be pressed. After the resting values have been determined, the microcontroller stores the maximum values for each direction. In addition, various possible value combinations are saved for the opposite strain gauge, which are later used for plausibility checks (see Section 3.2 Plausibility check). After determining all the values mentioned, multiplication factors are calculated in order to generate homogeneous output signals (see Figure 9) from the inhomogeneous input signals (see Figure 8). An error will be generated if the resting values fall outside of a certain range (theoretical resting value ±100). This value window is used to eliminate manufacturing tolerances. 

**Figure 11:**
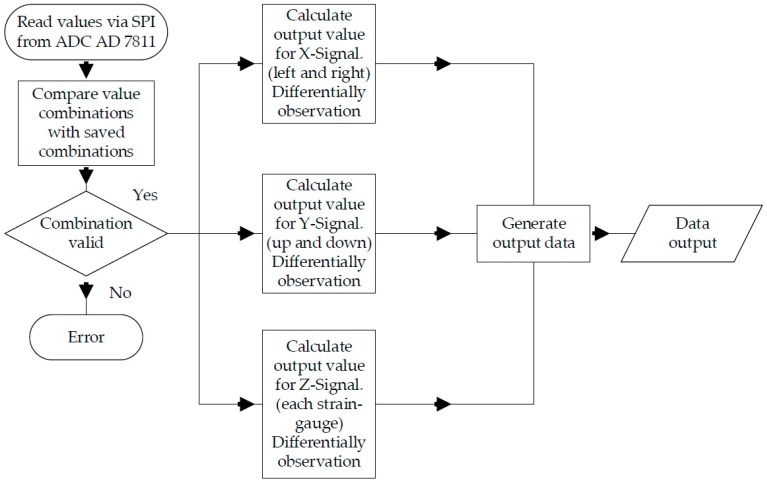
Schematic of sensor software under normal use.

During normal use (see Figure 11), the ADC’s data are read via SPI. Afterward, a plausibility check is performed. In doing so, the values of two strain gauges opposite each other are combined. Theses pairs of values have to match the value pairs stored during the calibration process. This way, a very large portion of all the possible errors can be identified. If there is an error, the microcontroller sends neutral values to the systems that are connected to it and a separate error notification is sent via I/O pin.

## 4. Results and Discussion

The strain gauge disc described here is in a fully functional prototype stage. The main challenge was the execution of the movement carrier. First, the movement carrier was manufactured using aluminum and steel. Despite the fact that the strain gauges were designed for aluminum and steel, the test series could not be completed in a climate-controlled cabinet. The maximum deviation for aluminum was ±5 and for steel it was ±8 (measured using a 10-bit ADC). During the climate test, the strain gauge disc was subjected to a temperature range of −30 °C to +80 °C in a climate cabinet. During the first test phase (see Table 5) the SGD was not deflected. 

The test period lasted a total of 8 h. The maximum temperature-based deviation of resting values of ±1 could only be attained with a movement carrier made of CFRP. A static deflection of the SGD during the second test (see Table 6) was conducted using a clamp (test period of 8 h). The deviations are within a maximum ±1 (measured with a 10-bit ADC). 

An additional series of tests examined the movement carrier’s reset accuracy (see Table 7) after moving the SGD in all directions with varying degrees of force.

The results of the stress test show that the movement carrier’s resting values deviate by a maximum of ±1 under different loads. The aluminum version had a deviation of ±6 whereas steel was ±4. Because deviations due to temperature and deviations after strain can occur simultaneously, a maximum deviation of ±2 needs to be accounted for. In order to counteract this problem, the strain gauges values have to deviate from the resting values by at least ±4, otherwise the downstream systems will not perform any actions. Tests under laboratory conditions were successfully completed. During the tests, the sensor was used as a substitute for a mouse and to operate an EPW [21]. As long as a physically disabled patient still has some type of physical capability [22] etc.) he or she should be able to use this sensor. If a patient’s symptoms change, the SGD can immediately be adapted to the user’s new range of strength and motion (without the help of service staff). This learning process takes a maximum of 10 s. The weight of the upper part of the casing can cause the SGD to vibrate when a certain oscillation frequency acts on the sensor. In laboratory tests, the resonance frequency was approximately 120 Hz. As a result the system might begin to vibrate. This rare case can also occur with conventional joysticks, but hardly occurs under normal conditions. In order to prevent this unlikely situation, we poured additional cross linking silicon-rubber underneath the CFRP carrier (version 2), which acts as a vibration damper. Owing to the design, the force to be exerted increases exponentially with the deflection causing a natural force feedback. This positive effect can be explained as follows: because the movement carrier can only be moved a few millimeters (3.6 mm) in each direction, users experience the system as being rigid. When a rigid system is subjected to an input force, the skin feels pressure, which is proportional to the input force. Users reported a very soothing effect compared to conventional joysticks. Conventional joysticks are usually equipped with springs. A proportional increase in counterforce is only minimal with this design. Without seeing a conventional joystick, the user cannot determine the relationship between the applied force and the actual deflection. 

## 5. Conclusions

This sensor provides a new input opportunity for spastic patients. Injuries or unintentional stops in operation due to sudden spastic episodes can be largely avoided. Used in combination with other recent developments cited in the introduction, this sensor could improve the human-machine interface for spastic conditions. Owing to the SGD’s simple design, a high cost of production is not expected. Especially in countries with poor health systems, the strain gauge disc is highly beneficial since no costs or only minor follow-up costs are to be expected even if the SGD would be used by another user with a different disease pattern. 

## Figures and Tables

**Table 1:** Selected measurements with different materials and versions. (only $X_{Right}$ is shown).

Test Series; Force (N) Disc Diameter of 120 mm Mat. (Material)	0	0.05	0.1	0.2	0.5	1	2	3	5	10	20	25
Mat. 1 CFRP 0.45 mm non silicon rubber	532	538	545	561	592	650	760	890	1024	1024	1024	1024
Mat. 2 CFRP 0.90 mm non silicon rubber	535	537	540	551	569	622	698	810	903	1024	1024	1024
Mat. 3 CFRP 1.30 mm non silicon rubber	510	511	513	516	529	550	611	675	776	895	1024	1024
Mat. 4 CFRP 2.00 mm non silicon rubber	521	521	521	521	522	528	549	607	673	790	897	1024
Mat. 5 CFRP 0.90 mm silicon rubber shore 20	505	506	507	511	558	611	670	796	881	982	1024	1024
Mat. 6 CFRP 0.45 mm silicon rubber shore 30	535	537	542	561	614	666	791	873	970	1024	1024	1024

**Table 2:** Explanation of materials used in Table 1.

Material	CFRP Thickness	Silicon Rubber	Shore Hardness
Material 1	0.45 mm	no	none
Material 2	0.90 mm	no	none
Material 3	1.30 mm	no	none
Material 4	2.00 mm	no	none
Material 5	0.90 mm	yes	20
Material 6	0.45 mm	yes	30

**Table 3:** Patient measurements and applied force.

Direction	Maximum Values	Approximate Force
forward	510	18.4 N
backward	109	2.1 N
left	390	11 N
right	430	14.2 N

**Table 4:** Maximum patient measurements and calculated output values.

Direction to	Maximum	Factor	Calculated Output Values
forward	510	2007	1024
back	109	9394	1024
left	390	2626	1024
right	430	2381	1024

**Table 5:** CFRP movement carrier, resting values in climate-controlled cabinet.

Temperature °C	Resting Values Forward	Resting Values Backward	Resting Values Right	Resting Values Left
−30	509	499	502	504
−20	509	499	502	504
−10	510	499	502	504
0	510	499	502	504
10	510	499	502	504
20	510	499	502	504
30	510	499	502	504
40	510	499	502	504
60	511	500	502	504
70	511	500	502	505
80	510	500	502	505

**Table 6:** Fixed deflection of the SGD forward and to the right in a climate-controlled cabinet.

Temperature °C	Resting Values Forward	Resting Values Backward	Resting Values Right	Resting Values Left
−30	829	182	769	231
−20	829	182	769	231
−10	829	182	770	231
0	829	182	770	231
10	829	182	770	231
20	829	182	770	231
30	829	183	770	231
40	829	183	770	231
60	829	183	770	231
70	830	183	770	231
80	829	183	771	231

**Table 7:** Resting position after strain (temperature 21 °C).

Strain (N)	Resting Values Forward	Resting Values Backward	Resting Values Right	Resting Values Left
Resting Values	510	499	502	504
forward 25	510	499	502	504
forward 100	510	499	502	504
forward 500	511	499	502	504
forward 1400	511	499	502	504
backward 25	510	499	502	504
backward 100	510	499	502	504
backward 500	509	498	502	504
backward 1400	509	498	502	504
right 25	510	499	502	504
right 100	510	499	502	504
right 500	510	499	503	503
right 1400	510	499	503	503
left 25	510	499	502	504
left 100	510	499	502	505
left 500	510	499	502	505
left 1400	510	499	502	505
